# Spatial correlation network structure of energy-environment efficiency and its driving factors: a case study of the Yangtze River Delta Urban Agglomeration

**DOI:** 10.1038/s41598-023-47370-8

**Published:** 2023-11-27

**Authors:** Shucheng Liu, Jie Yuan

**Affiliations:** 1 School of Statistics and Mathematics, Zhejiang Gongshang University, Hangzhou, China; 2 School of Public Finance and Taxation, Zhejiang University of Finance and Economics, Hangzhou, China

**Keywords:** Environmental sciences, Environmental social sciences

## Abstract

Improving energy-environment efficiency is not only a requirement for constructing China’s ecological civilization but also inevitable for achieving sustainable economic and social development. Studies on energy-environment efficiency based on relational data and network perspectives are limited, which hinders the development of collaborative regional emission reduction activities. This study uses the SBM-Undesirable model to measure the energy-environment efficiency of the Yangtze River Delta Urban Agglomeration from 2010 to 2020, adopts a modified gravity model and social network analysis to reveal the structural characteristics of its spatial correlation network, and explores its driving factors through the QAP method. The study found (1) an overall upward trend in energy-environment efficiency but with problems of uneven development. (2) The spatial correlation of energy-environment efficiency shows a complex network structure, with increasing network correlation and strong network stability; the network can be divided into four plates: net benefit, net overflow, two-way spillover, and agent. (3) Differences in industrial structure, environmental regulation, economic development, and technological innovation significantly impact the formation of spatial correlation network of energy-environment efficiency. This study provides a reference for the construction of a cross-regional synergistic mechanism to improve energy-environment efficiency.

## Introduction

Energy is a global and strategic issue that affects the development of human society, international political and economic landscape, and climate change and environmental pollution^1^. China is currently the world’s largest energy producer, consumer, and emitter of CO_2_. Reducing energy intensity and carbon emissions is an important goal of China’s energy development strategy^2^. The energy problem arises from the issue of energy use, thus improving energy efficiency is the key. Recently, the Chinese government has issued major programmatic documents such as the Opinions on Accelerating the Construction of Ecological Civilization and the Overall Programme for the Reform of the Ecological Civilization System, aiming to establish an economical, circular, and efficient resource utilization system, comprehensively improve energy-environment efficiency, and achieve green economic development.

Energy efficiency is defined as the amount of energy consumed that contributes to the maintenance and promotion of sustainable human development^3^. Environmental efficiency mandates that the intensity of environmental impacts and resource use be adapted to the planet’s carrying capacity to balance the ecological and economic benefits of economic activities^4^. It is a central expression of the goal of sustainable development. Study on energy and environmental efficiency focuses on the enterprise, industry, regional, and national levels^5–7^. The core idea of both energy and environmental efficiency is to create higher social value with less resource consumption and less environmental impact, then energy-environment efficiency can be defined as minimizing the impact of energy development and use on ecosystems while promoting economic growth and enhancing social welfare^8,9^. Thus, energy-environment efficiency studies are both environmental and energy efficiency evaluations with a focus on energy resources and environmental factors, respectively. Improving energy-environment efficiency is the essential requirement of developing low-carbon economy and the key to realize the transformation of economic development from high energy consumption, high carbon and high pollution to low energy consumption, low carbon and low pollution.

With comprehensive promotion of regional coordinated development strategies and the rapid development of transportation and information networks, there is accelerated flow of factors such as labor, capital, technology, and products between regions. The spatial association of industry^10^, finance^11^ and consumption^12^ has broken the geographical proximity limit and formed a spatial correlation network. As a product of economic activity, the spatial effects of energy consumption also transcend geographical proximity and gradually assume the characteristics of a spatial network^13^. In this context, the study of the spatial relationship of energy-environment efficiency should also be further deepened to identify new trends and characteristics under the coordinated regional development strategy and provide more useful guidance for optimizing its spatial pattern.

Although academics have explored the spatial relationship of energy efficiency and analyzed its spatial correlation, spillover effects, and convergence characteristics, there are still some limitations to these studies. On the one hand, studies have only considered the spatial effects of geographical proximity, lacking a holistic approach and ignoring the possible impact of non-neighboring areas on energy-environment efficiency; on the other hand, studies are mainly based on attribute data rather than relationship data, which can only reflect the current situation of energy-environment efficiency in each region but cannot accurately portray the network structure characteristics of spatially linked energy-environment efficiency relationships. Thus, this study delves into the spatial correlation network structure of energy-environment efficiency in the Yangtze River Delta urban agglomeration and its driving factors based on relational data and spatial network perspectives.

The main contributions of this study are as follows: (1) Based on a modified gravity model, a spatial correlation network of energy-environment efficiency among cities is constructed, and the structural characteristics and evolutionary trends of the spatial network are explored using social network analysis (SNA), focusing on the specific position and role of each city in the spatial correlation network. (2) The quadratic assignment procedure (QAP) is used to explore the drivers of the spatial correlation network of energy-environment efficiency, effectively circumventing the problem of multicollinearity among variables that leads to biased statistical test results. This study opens a new perspective on the study of energy-environment efficiency and helps provide reference for developing global and regionally linked energy efficiency improvement policies.

The remainder of this study is organized as follows. Section "Literature review" presents a literature review. Section "Study area" introduces the research area. Section "Methodology and materials" provides a description of the methodology and the data. Section "Results" analyzes the empirical results. Section "Conclusion and discussion" presents the main conclusions and discussion.

## Literature review

### Measurement of energy-environment efficiency

Academic measures of energy efficiency have been developed from both single- and total factor perspectives. As single-factor energy efficiency measures overlook energy as a productive input that cannot contribute to economic growth on its own^14^, there is a growing trend among scholars to comprehensively measure energy efficiency within a total factor framework. The main methods for evaluating total factor energy efficiency include data envelopment analysis (DEA) and stochastic frontier analysis, which is dominated by DEA, with models including Constant Return to Scale, Variable Return to Scale, three-stage DEA, Malmquist index, and super-efficiency DEA^15–18^ pioneered the use of total factor energy efficiency indicators to analyses energy-environment efficiency but did not consider the impact of undesired outputs. Mandal^19^ and Wu et al.^20^ showed that the traditional DEA models that ignore undesired outputs would lead to biased measures of energy efficiency. Wang et al.^21^ confirmed the existence of this bias in a subsequent study by comparing energy efficiency with and without undesired outputs. Li^22^ measured regional energy efficiency in China from both static and dynamic perspectives based on a total factor and multiple output frameworks and found that neglecting undesired outputs would result in considerable overestimation of energy efficiency.

With academics increasingly concerned about environmental pollution and ecological damage in the energy consumption process, slacks-based measure (SBM) models that consider undesired outputs are widely used in total factor energy efficiency evaluation^23,24^; however, different scholars choose different indicators and methodological treatment of undesirable outputs. Gokgoz and Erkul^25^ argued that the energy use process mainly causes atmospheric pollution, with the use of fossil fuels accounting for about 70% of CO_2_ emissions, 90% of SO_2_ emissions, and 67% of NO_2_ emissions in the air; therefore, industrial emissions were chosen as an indicator of pollution. Fang et al.^26^ used the entropy method to combine industrial wastewater emissions, industrial emissions, and other indicators into a pollution index as a nondesired output indicator, and Fang et al.^27^ merged desired output GDP and nondesired output CO_2_ emissions into a combined output indicator. In this study, the SBM-Undesirable model was constructed to measure energy-environment efficiency using the entropy method to integrate CO_2_ emissions, industrial wastewater emissions, industrial SO_2_ emissions, and industrial soot emissions as undesirable output indicators.

### Spatial analysis of energy-environment efficiency

As spatial factors and the development of spatial econometric analysis techniques have attracted academics, several studies have focused on the spatial correlation of energy-environment efficiency. Existing studies have focused on the spatial correlation and spatiotemporal convergence dimensions of energy efficiency, mainly at the provincial and industry levels^28–30^ confirmed a significant spatial correlation and growing dependence between regional energy efficiency. Song et al.^31^ studied the spatial pattern and effects of energy eco-efficiency in China and found a significant spatial spillover effect of energy eco-efficiency between neighboring regions. Shi and Zhang^32^ used the coefficient of variation to examine the convergence of energy efficiency in China as a whole, with the western region showing divergence, eastern showing convergence, and central showing convergence toward the east. Wang et al.^33^ found that a narrowing interprovincial energy efficiency gap in China and some convergence in energy efficiency. However, the existing studies mainly focus on the development and relevance of energy-environment efficiency in various regions, while ignoring the network structure characteristics of the spatial correlation of energy-environment efficiency.

Generally, significant regional differences in energy-environment efficiency result from the combination of economic structure, technology level, and energy consumption patterns^34^, while the spatial association of energy-environment efficiency is driven by both government intervention and market mechanisms, resulting in a systematic and complex network structure^35^. Therefore, reexamining the spatiotemporal relationship of energy-environment efficiency and its improvement strategies from a network perspective is trending in the field of sustainable development^36,37^. In this context, it is theoretically and practically imperative to scientifically measure the urban energy-environment efficiency, deeply explore the structure and driving factors of the spatial correlation network of energy-environment efficiency in the Yangtze River Delta urban agglomeration, and clarify the status and role of each region in the spatial correlation network to build a cross-regional synergistic mechanism for improving energy-environment efficiency.

## Study area

**Figure 1:**
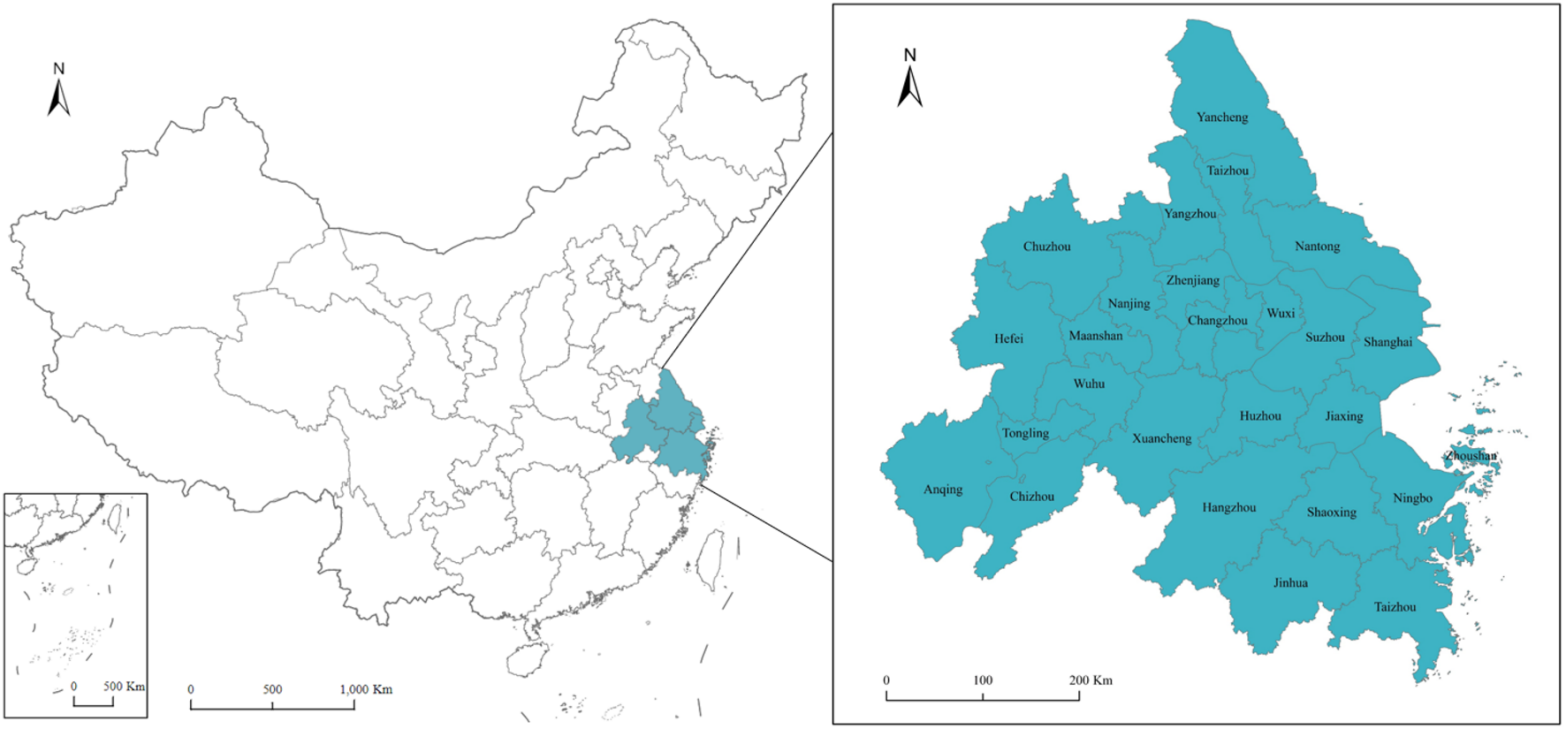
Geographical location of the Yangtze River Delta Urban Agglomeration. Produced based on the standard map number GS (2019)1719 downloaded from the standard map service website of the Ministry of Natural Resources of China.

The Yangtze River Delta Urban Agglomeration is located at the important intersection of the “One Belt and One Road” and the Yangtze River Economic Belt, covering 26 prefecture-level cities. The specific area is shown in Fig. 1. The Yangtze River Delta Urban Agglomeration accounts for only 2.3% of China’s regional area and 10% of the total population, contributing about 20% of the GDP, and the total energy consumption accounts for about 15% of the country’s total energy consumption (National Bureau of Statistics, 2021). This region has the strongest economic development in China. According to the Chinese government’s “14th Five-Year Plan” target, by the end of 2025 (compared with 2020), the energy consumption and carbon emissions per unit of GDP will be reduced by 13.5% and 10%, respectively. These environmental protection-related policies will inevitably have a fundamental impact on China’s energy-environment efficiency^38^. The Yangtze River Delta region has a pivotal strategic position in the overall situation of China’s modernization drive, and the coordinated development of this region is an important breakthrough and focus to achieve sustainable economic development. With the formation of the transportation network and the flow of economic factors, the spatial connection of the Yangtze River Delta Urban Agglomeration has been continuously strengthened, and the network characteristics have become increasingly obvious. Thus, it is imperative to study the spatial network structure characteristics and driving factors of its energy-environment efficiency to provide guidance for building a new type of urban agglomeration of ecological civilization.

## Methodology and materials

### Calculation of energy-environment efficiency

The SBM model is based on DEA method and is proposed to address the bias and influence caused by radial and angular selection, and can effectively solve the problem of inability to compare decision units in practical applications^39^. In this study, energy-environment efficiency is defined as the energy efficiency when environmental constraints are considered under the total factor framework, and the SBM-Undesirable model is used to measure it. The model expression is as follows:

1 $$ \begin{array}{*{20}c} {\begin{array}{*{20}c} {\begin{array}{*{20}c} {\min \rho_{SE} = \frac{{1 - \frac{1}{m}\sum\limits_{i = 1}^{m} {s_{i}^{ - } /x_{ik} } }}{{1 + \frac{1}{{s_{1} + s_{2} }}\left( {\sum\limits_{r = 1}^{{s_{1} }} {s_{r}^{ + } /y_{rk} } + \sum\limits_{t = 1}^{{s_{2} }} {s_{t}^{z - } /z_{tk} } } \right)}}} \\ {s.t.} \\ \end{array} } \\ {\sum\limits_{j = 1,j \ne k}^{n} {x_{ij} \gamma_{j} - s_{i}^{ - } \le x_{ik} } } \\ \begin{gathered} \sum\limits_{j = 1,j \ne k}^{n} {y_{rj} \gamma_{j} + s_{r}^{ + } \ge y_{rk} } \hfill \\ \sum\limits_{j = 1,j \ne k}^{n} {z_{rj} \gamma_{j} + s_{t}^{z - } \le z_{rk} } \hfill \\ \end{gathered} \\ \end{array} } \\ {\gamma ,s^{ - } ,s^{ + } ,s^{z - } \ge 0} \\ {i = 1,2, \ldots ,q;j = 1,2, \ldots ,n(j \ne k)} \\ \end{array} $$

where $\rho_{SE}$ is the efficiency value, *X* is the input variable, *y* and *z* respectively denote the expected and undesired output variables, and *m* represents the number of input indicators. In this model, $s_{1}$ and $s_{2}$ respectively signify the number of indicators of expected and undesired output and *k* denotes the production period. Additionally, *i*, *r,* and *t* represent the decision-making units of input, desired output, and undesired output, respectively. Further, $s^{ - }$, $s^{ + }$, and $s^{z - }$ indicate the slack variables of input, desired output, and undesired output, respectively with $\gamma$ as the weight vector. The larger the $\rho_{SE}$ value, the higher the efficiency. $\rho_{SE}$ = 1 indicates an effective decision-making unit; $\rho_{SE}$ < 1 signifies a relatively ineffective decision-making unit and indicates efficiency loss.

According to the production function in the economic growth theory, energy consumption, capital stock, and labor are selected as input indicators, GDP as expected output indicators, and pollution index as undesirable output indicators. The description of the indicators is shown in Table 1.

**Table 1:** Input–output indicator system of energy-environment efficiency.

Variable	Indicator	Description
Inputs	Energy consumption (10^4^ t SCE)	Using the consumption of natural gas, liquefied petroleum gas, and electricity consumption of the whole society to convert the consumption
	Capital stock (10^4^ Yuan)	Estimated using the perpetual inventory method, adjusted based on the provincial fixed capital investment price index with 2010 as the base period
	Labor force (10^4^ people)	The total number of labor employees is obtained by adding up the number of employees in urban units, and the number of private and individual employees in urban areas at the end of the year
Desirable outputs	GDP (10^4^ Yuan)	Adjusted to GDP at comparable prices using 2010 as the base period
Undesirable outputs	Pollution index	Comprehensive measurement of CO_2_, industrial wastewater, industrial sulfur dioxide, and industrial smoke and dust emissions using the entropy method

### Analysis of spatial correlation network structure

#### Construction of spatial correlation network

The determination of relationships is the key to network analysis, and studies have mainly used gravity models and vector autoregressive (VAR) Granger causality tests to determine the relationships between variables^40,41^. As the VAR model cannot portray the evolutionary trend of the spatial correlation network and is too sensitive to the lag order, which fairly reduces the accuracy of the spillover relationship portrayal^38^. This study uses a modified gravity model to measure the relationship of urban energy-environment efficiency to construct a spatial correlation network. The specific formula is as follows:

2 $$ Q_{ij} = \frac{{G_{i} }}{{G_{i} + G_{j} }} \times \frac{{EE_{i} \times EE_{j} }}{{\frac{{D_{ij}^{2} }}{{(ag_{i} - ag_{j} )^{2} }}}} $$

where *Q*_*ij*_ represents the gravitational strength of the energy-environment efficiency between city *i* and city *j*; *EE*_*i*_ and *EE*_*j*_ represent the energy-environment efficiency of city *i* and city *j*; *D*_*ij*_ represents the geographic spatial distance (latitude and longitude distance) between city *i* and city *j*; *G*_*i*_, *G*_*j*_, *ag*_*i*_, and *ag*_*j*_ represent the GDP and per capita GDP of city *i* and city *j*, respectively.

Using the gravity model to construct the gravity matrix of the urban energy-environment efficiency as the basis for the network structure analysis. The average value of each row in the matrix is used as the critical value, and the gravitational value higher than the critical value is recorded as 1, indicating a spatial overflow of energy-environment efficiency for the city in this row; the gravitational value below the critical value is recorded as 0, indicating no spatial overflow.

#### Analysis of network structure characteristics

SNA is a scientific method for analyzing relational networks based on relational data. Referring to studies on spatial correlation networks^42–44^, this study explores the network structure characteristics of energy-environment efficiency from three aspects: overall, node, and cluster structures.

1. Overall network structure characteristics. This feature is analyzed using five indicators: number of relationships, density, correlation, rank, and efficiency (Table 2). The number of network relationships and network density are used to reflect the strength of correlation of each node in the spatial correlation network; the greater the number of network relationships, the greater the network density, indicating a stronger correlation of energy-environment efficiency. The network correlation is used to reflect the robustness of the spatial correlation network; a value of 1 indicates that the energy-environment efficiency has a spatial network effect and the network is robust. The network rank is used to reflect the status difference of urban energy-environment efficiency; the higher the rank, the greater the rank difference in the network. The network efficiency is used to reflect the stability of the spatial correlation network; the lower the network efficiency, the more stable the network.
2. Node network structure characteristics. This feature is analyzed using three indicators: degree, closeness and intermediary centrality (Table 2). A higher degree centrality of a city means that the city is closer to the center of the spatial correlation network and has a stronger effect on the rest of the network nodes. The higher the closeness centrality of a city, the closer the city is to other cities in the spatial association network. A higher intermediary centrality of a city means that the city has a stronger control and regulation effect on the energy-environment efficiency of other cities in the spatial correlation network.
3. Spatial clustering structure characteristics. The block model is used to conduct cluster analysis on the spatial correlation network to explore the roles and functions of each plate in the network. This study classifies the spatial correlation network of energy-environment efficiency into four plates: net benefit, net overflow, two-way spillover, and agent. The attribute of the plate is judged by the number of incoming and outgoing relations and the number of members within the plate (Table 3).

**Table 2:** Characteristic indicators of energy-environment efficiency network.

Indicator	Calculation method	Variable meaning
Network density	*D* = *L*/[*N* (*N* − 1)]	*N* and *L* denote the total number of cities and the actual number of intercity associations, respectively
Network correlation	*C* = 1 − 2* V*/[*N* (*N* − 1)]	*V* denotes the number of unreachable point logarithms
Network rank	*R* = 1 − *K*/max*K*	*K* and max*K* denote the actual number of symmetrically accessible point pairs and the maximum number of symmetrically accessible point pairs, respectively
Network efficiency	*E* = 1 − *M*/max*M*	*M* and max*M* denote the number of redundant links between cities and the maximum possible number of redundant links, respectively
Degree centrality	*C*_*RD*_ = *n*/(*N* − 1)	*n* indicates the number of other cities in the network that are connected to a particular city
Closeness centrality	$C_{AP} = \frac{{\sum\nolimits_{j = 1}^{n} {d_{ij} } }}{N - 1}$	*d*_*ij*_ denotes the shortcut distance between city *i* and city *j*
Intermediary centrality	$C_{RB} = \frac{{2\sum\nolimits_{j = 1}^{N} {\sum\nolimits_{k = 1}^{N} {b_{jk} (i)} } }}{{N^{2} - 3N + 2}}$	$b_{jk} (i) = g_{jk} (i)/g_{jk}$ denotes the ratio of the number of shortcuts passing through city *i* and connecting city *j* with city *k* to the total number of shortcuts between these two points

**Table 3:** Classification of energy-environment efficiency plate attributes in the block model.

Proportion of relationships within the location	Proportion of relationships received by this position	
	≈ 0	> 0
≥ (*g*_*k*_ − 1)/(*g* − 1)	Two-way overflow plate	Net benefit plate
< (*g*_*k*_ − 1)/(*g* − 1)	Net spillover plate	Agent plate

*g*_*k*_ is the number of members within the block, *g* is the number of members within the network relationship.

### Analysis of driving factors of spatial correlation network

The QAP method does not require the assumption of independence and normal distribution. The results are more robust when using the QAP method to analyze relational data, as the variables involved in the study are all relational and there may be multicollinearity between variables, and it is difficult to determine whether the disturbance terms follow a normal distribution^45^. To further reveal the intrinsic drivers of the evolution of spatial correlation network of energy-environment efficiency, the following econometric model was constructed:

3 $$ EQ = f(IS,ECS,ER,ED,OU,TI) $$

Numerous factors, mainly involving structural, actor, and external shock, affect energy-environment efficiency. Structural factors include industrial, property rights, and energy consumption; actor factors include government environmental regulation and enterprise environmental self-control ability; and external shock factors include the level of economic development, opening up, and technological progress. The potential drivers of the spatial correlation of energy-environment efficiency are selected in the study as follows: (1) Industrial structure differences (*IS*). Energy-environment efficiency is closely related to the scale of the tertiary sector, and the rapid development of the tertiary sector is conducive to strengthening the spatial correlation of energy-environment efficiency^46^. (2) Energy consumption structure differences (*ECS*). The differences in energy consumption structure are mainly reflected in the use of fossil fuels. The differences in energy-environment efficiency under different energy consumption structures are large, and such differences may encourage the formation of spatial correlations in energy-environment efficiency^47^. (3) Environmental regulation differences (*ER*). Firms in regions with stronger environmental regulations tend to move to regions with less intense environmental regulations, causing the movement of production factors and thus enhancing the correlation effect of energy-environment efficiency. (4) Economic development differences (*ED*). Under the market mechanism, factors of production probably move between regions with similar levels of economic development, and thus energy-environment efficiency might be correlated^48^. (5) Opening up differences (*OU*). External openness can lead to international mobility of production factors, which is unfavorable to intra-regional cooperation in production activities, thus weakening the correlation effect of energy-environment efficiency^49^. (6) Technological innovation differences (*TI*). Differences in the level of technological innovation can hinder the transfer and absorption of new technologies between regions, which impedes the development of energy-environment efficiency linkages. Referring to the general practice of scholars^50^, select the data of the last year (2020) of the sample period and use the revised gravity model to construct a regional difference matrix. The selection of specific indicators is shown in Table 4.

**Table 4:** Variable descriptions in the QAP method.

Variable	Description	Indicator
*EQ*	Energy-environment efficiency differences	Energy-environment efficiency
*IS*	Industrial structure differences	Proportion of tertiary sector value added in GDP (%)
*ECS*	Energy consumption structure differences	Proportion of coal consumption in total energy consumption (%)
*ER*	Environmental regulation differences	Proportion of government fiscal expenditure in GDP (%)
*ED*	Economic development differences	Adjusted to GDP per capita at comparable prices using 2010 as the base period (Yuan)
*OU*	Opening up differences	Proportion of total import and export trade in GDP (%)
*TI*	Technological innovation differences	Proportion of R&D expenditure in government fiscal expenditure (%)

### Data source

This study focuses on 26 prefecture-level cities in the Yangtze River Delta Urban Agglomeration, and the time span is set from 2010 to 2020. The research data comes from the China Statistical Yearbook and the 26-city Statistical Yearbook over the years. Currently, the main energy statistics at the prefecture-level city level include natural gas, liquefied petroleum gas, and electricity consumption of the whole society; therefore, these three energy consumption data are converted into total energy consumption. According to the “reference coefficient of standard coal for various energy sources,” it is uniformly converted into standard coal consumption. The reference coefficients of standard coal for natural gas, liquefied petroleum gas, and electricity are 1.330 kg SCT/m^3^, 1.714 kg SCT/kg, and 0.123 kg SCT/kW h, respectively. The carbon emission data are based on the guidance method of IPCC^51^ to measure the total amount of CO_2_ produced by the combustion of various fossil fuels. The city distance data are calculated by the distance function of ArcGIS software, and the vector base map data are obtained from the standard map of the Ministry of Natural Resources (GS(2019)1719).

## Results

### Spatiotemporal evolution of energy-environment efficiency

**Figure 2:**
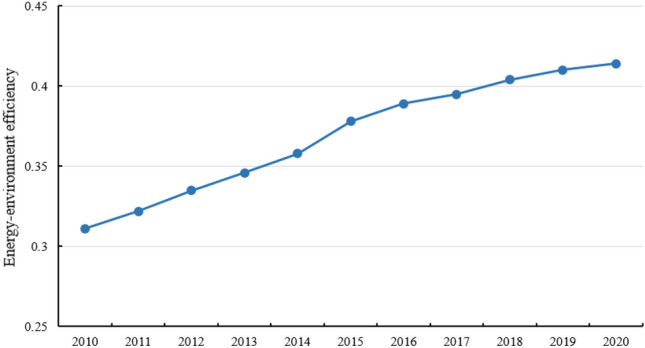
Time-series evolution of energy-environment efficiency.

**Figure 3:**
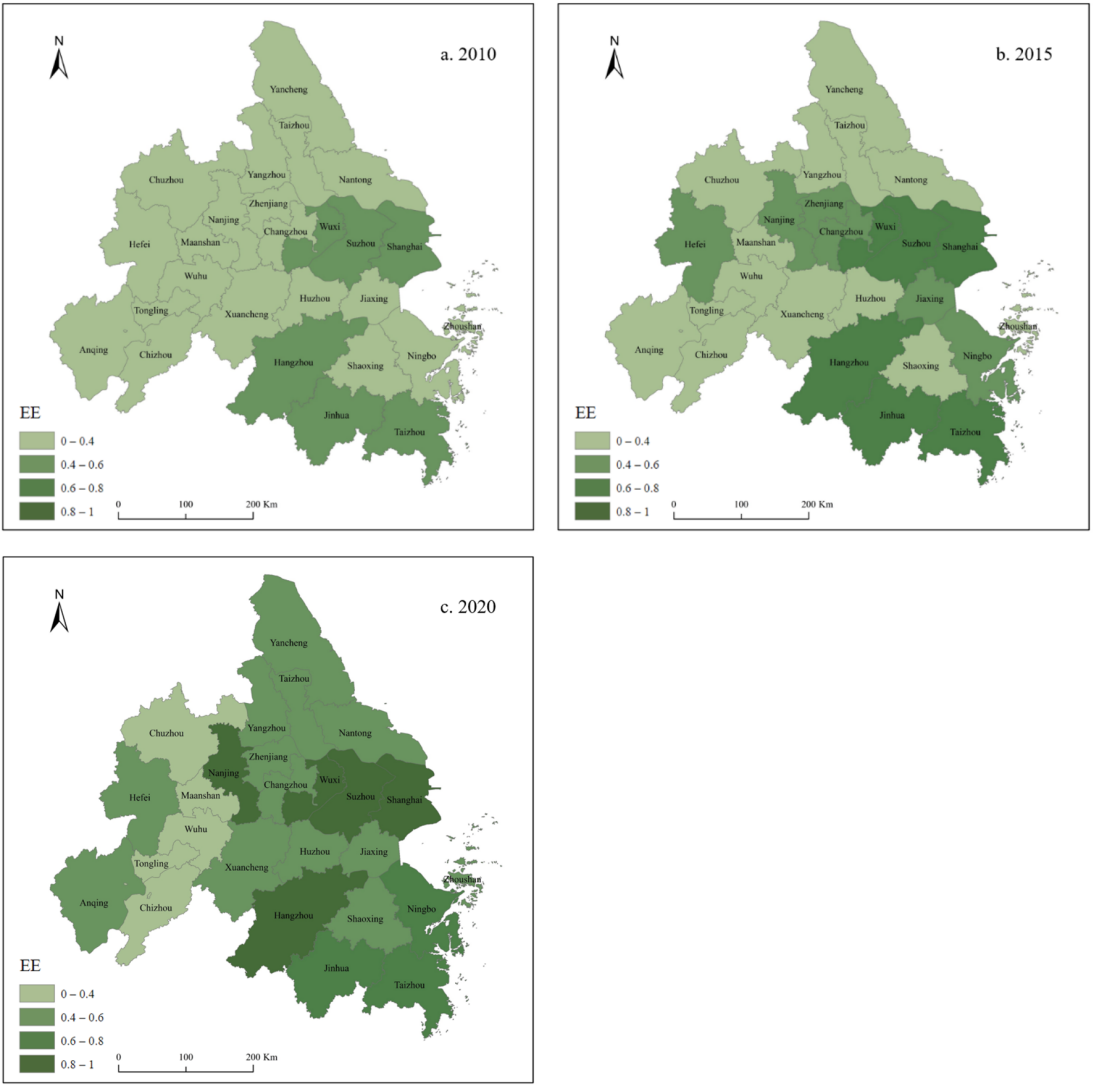
Spatial distribution of energy-environment efficiency. Authors used Arcgis 10.8 software to draw the map. https://www.arcgis.com/index.html.

The energy-environment efficiency of the Yangtze River Delta Urban Agglomeration from 2010 to 2020 was measured using the SBM-Undesirable model. Consistent with the “time-series evolution–spatial distribution” approach, the time-series evolution of energy-environment efficiency (Fig. 2) and the spatial distribution patterns (Fig. 3) were investigated.

In terms of time-series evolutionary characteristics (Fig. 2), the Yangtze River Delta Urban Agglomeration shows an overall upward trend in energy-environment efficiency. The average value of energy-environment efficiency rose from 0.381 in 2010 to 0.571 in 2020, an increase of 49.87%, indicating considerable green development of the Yangtze River Delta Urban Agglomeration over the past 11 years, mainly due to the return of ecological culture and the increasing importance of the construction of ecological civilization. Under environmental constraints, the same energy input reaps more economic output, or relatively less environmental pollution emissions for the same input and output, reflecting the harmonious development of energy consumption and environmental protection. The underlying reasons are, on the one hand, improved economic output because of the increase in production efficiency under the development of technology, with the promotion and application of information technology, new industrial equipment, clean energy and other related technologies; on the other hand, with the implementation of high-quality development and ecological civilization construction strategy, under the guidance of policy and market green consumption, enterprises have focused more on energy saving and emission reduction, reducing environmental pollution.

In terms of spatial distribution characteristics (Fig. 3), the Yangtze River Delta Urban Agglomeration shows a stepped development pattern of “high in the east and low in the west,” with the general growth and obvious disparity in energy-environment efficiency of each city being its distinctive features. In 2010, cities with high energy-environment efficiency were mainly located in the eastern and southern regions, with no significant spatial differentiation. In 2015, high energy-environment efficiency showed a tendency to expand to the periphery, with cities in the east showing a marked increase, those in the center showing a slight improvement, and most in the west showing a lower energy-environment efficiency, with no significant change compared with 2010. In 2020, the regional gap in energy-environment efficiency converged because the western region is undergoing rapid industrialization, with rapidly increasing fossil energy consumption, with slow upgrading of the corresponding environmental protection measures and rough economic development; therefore, energy-environment efficiency remains low. Contrarily, the developed cities in the east have completed industrial restructuring and development model transformation, with more advanced environmental protection equipment and technologies, high energy utilization efficiency, and significant economic benefits. Overall, the energy-environment efficiency increased significantly from 2010 to 2020, the gap between cities gradually narrowed, and the spatial distribution continued to deepen into the central region, gradually tending to balanced development. The low-carbon development and green transformation in economically developed regions play an increasingly prominent role in achieving regional green development goals and leading the transformation of development models in the surrounding areas.

### Analysis of spatial correlation network structure of energy-environment efficiency

#### Overall network structure characteristics

**Figure 4:**
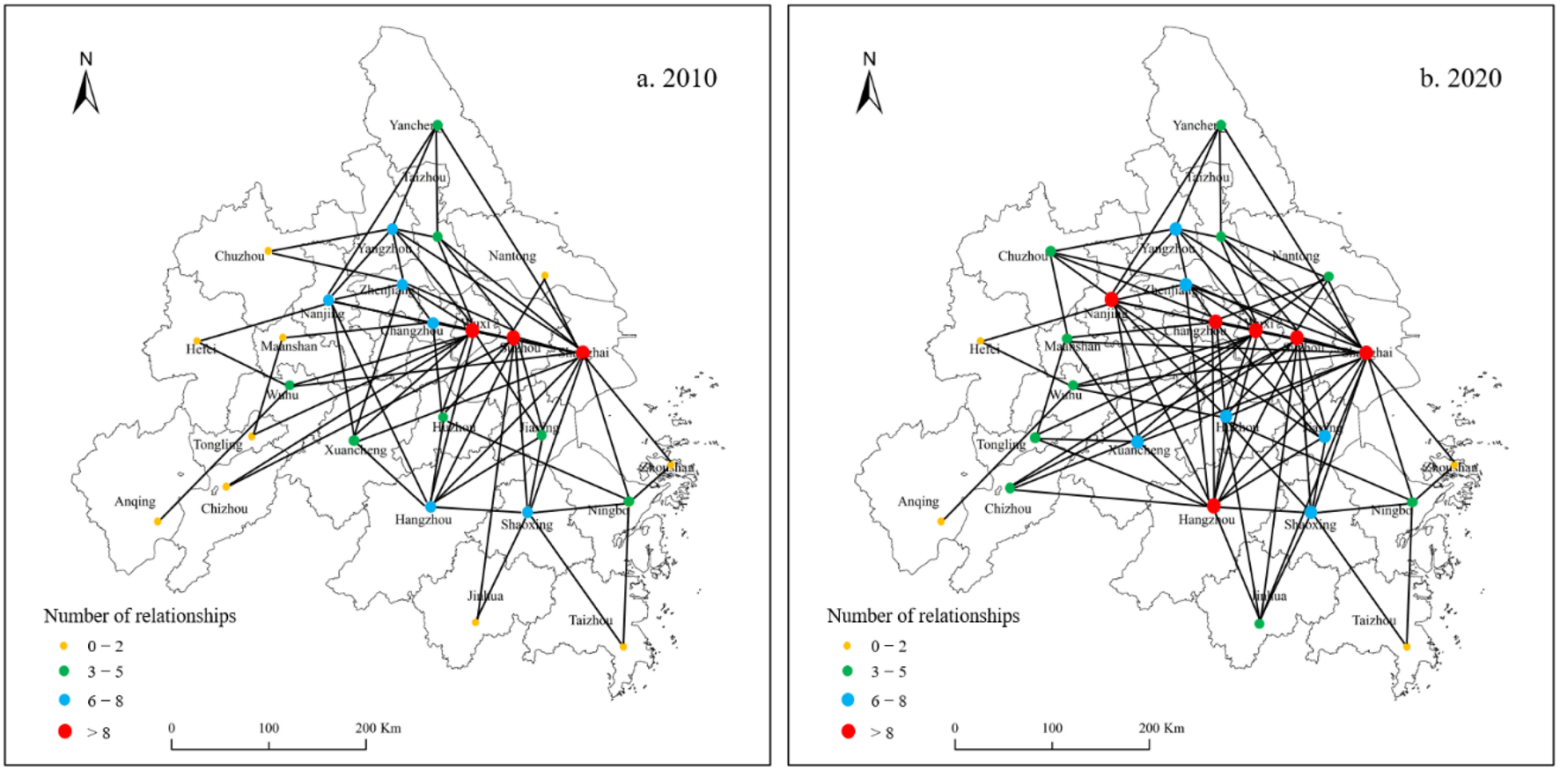
Spatial correlation network of energy-environment efficiency. Authors used Arcgis 10.8 software to draw the map. https://www.arcgis.com/index.html.

In this study, the gravity model is used to construct the spatial correlation matrix of energy-environment efficiency of the Yangtze River Delta Urban Agglomeration, and the spatial correlation network diagram of energy-environment efficiency is drawn using ArcGIS visualization tools (Fig. 4). The energy-environment efficiency of the Yangtze River Delta Urban Agglomeration has broken through the traditional spatial and geographical proximity spillover attributes, there are no isolated points in the network, and the overall feature of a complex spatial correlation network with multithreading and multiflow is presented.

The overall network structure characteristics indicators were further calculated using Ucinet 6.0 software (Table 5), which show the following:

1. The number of network relationships for energy-environment efficiency in the Yangtze River Delta Urban Agglomeration shows a fluctuating upward trend, rising from 84 in 2010 to 158 in 2020, an increase of 88.09%, with the maximum value being 160 in 2016. Network density also shows a fluctuating upward trend, rising from 0.141 in 2010 to 0.253 in 2020, an increase of 79.43%, with the maximum value is 0.254 in 2016. The spatial correlation strength of energy-environment efficiency has gradually increased, and the interaction between urban energy-environment efficiency has been strengthened because, on the one hand, the gradual improvement of the market economic system enables convenient circulation of production factors within the region, and on the other hand, the rapid development of transportation and information networks has strongly supported activities such as labor transfer, capital investment, and technology exchange between cities, strengthened industrial linkages between cities, and provided guarantees for the formation of spatial correlations in energy-environment efficiency. Although there is great improvement in the network relationship number of the energy-environment efficiency, a large gap still exists compared with the maximum possible relationship number of 650 (26 × 25) and its spatial relationship can be further improved.
2. From 2010 to 2020, the network correlation degree of energy-environment efficiency of the Yangtze River Delta Urban Agglomeration was 1, and each city is included in the spatial correlation network. This shows that the structure of the spatial correlation network is robust, the urban energy-environment efficiency is interconnected, and the spatial spillover effect of energy-environment efficiency has a wide range and is not limited to the neighborhood.
3. The network rank of energy-environment efficiency of the Yangtze River Delta Urban Agglomeration fluctuated greatly from 0.456 in 2010 to 0.265 in 2020, implying a decrease of 41.89%, the maximum value was 0.503 in 2012, and the minimum value is 0.192 in 2018; the overall showing a downward trend. This shows that the strict hierarchical structure within the network is gradually disintegrating, and the interconnection and impact of urban energy-environment efficiency are strengthened because under the influence of factors such as market, transportation, information, and policies, labor, capital, and technical elements that are coordinated in the region, which promotes the convergence of the energy-environment efficiency gap between cities.
4. The network efficiency of energy-environment efficiency in the Yangtze River Delta Urban Agglomeration showed a downward trend, from 0.835 in 2010 to 0.684 in 2020, a decrease of 18.08%, which implies a gradual enhancement of the network stability, improved the coordination of social and economic development, increased correlation between urban energy-environment efficiency, closer network, and improved stability.

**Table 5:** Overall network structure characteristics indicators of energy-environment efficiency from 2010 to 2020.

Year	Number of network relationships	Network density	Network correlation	Network rank	Network efficiency
2010	84	0.141	1	0.456	0.835
2011	89	0.150	1	0.498	0.823
2012	112	0.184	1	0.503	0.762
2013	127	0.213	1	0.322	0.741
2014	120	0.199	1	0.424	0.743
2015	142	0.235	1	0.281	0.706
2016	160	0.254	1	0.374	0.649
2017	149	0.241	1	0.234	0.684
2018	154	0.250	1	0.192	0.687
2019	147	0.239	1	0.293	0.690
2020	158	0.252	1	0.265	0.684

#### Node network structure characteristics

**Figure 5:**
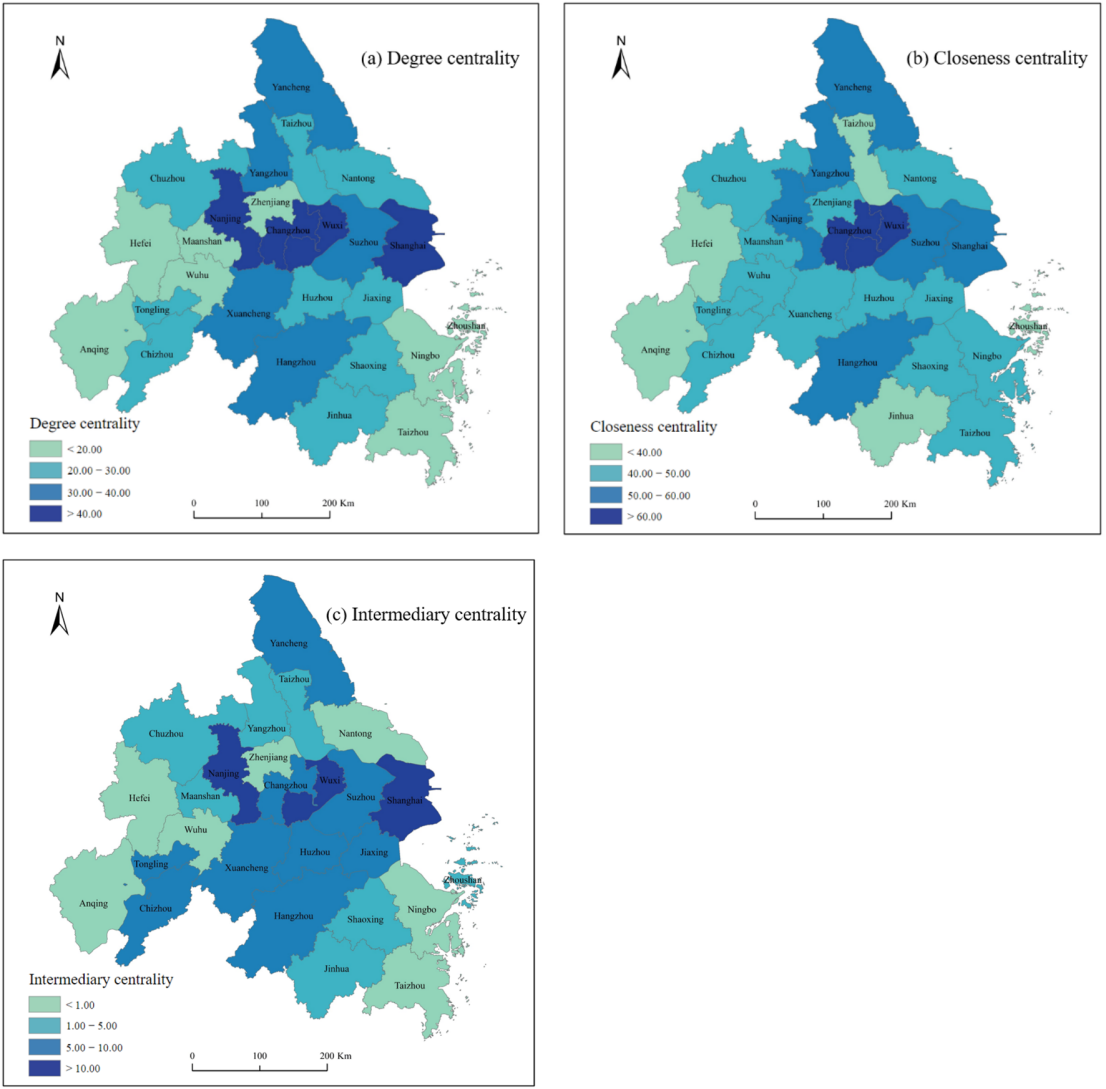
The spatial distribution of degree centrality, closeness centrality and intermediary centrality. Authors used Arcgis 10.8 software to draw the map. https://www.arcgis.com/index.html.

Using Ucinet 6.0 software to calculate the node structure characteristics of the spatial correlation network of energy-environment efficiency in the Yangtze River Delta Urban Agglomeration, Fig. 5 shows the calculation results in 2020.

1. In 2020, the mean degree centrality of the spatial correlation network of energy-environment efficiency is 35.27. The degree centrality of Shanghai, Nanjing, Wuxi, and Changzhou is higher than the average value, indicating that these cities are closer to the center of the network, have more connections with other cities, and play a key role in the formation and stable development of the overall network. The underlying reason is that these cities are economically developed regions, where advanced technology and well-developed transport networks significantly influence the economic output of other cities through technology transfer, capital investment, and labor attraction; simultaneously, these cities, with their dense populations and high per capita incomes, are the main consumption areas for the products. Therefore, their green consumption habits will have a reverse effect on the production operations in other cities, thus reducing environmental pollution. Anqing, Hefei, Wuhu, and Maanshan cities have a low degree centrality and are subordinates in the associated network.
2. The mean value of the closeness centrality of the spatial correlation network of energy-environment efficiency in 2020 was 51.80. The closeness centrality of Changzhou, Wuxi, Nanjing, and Suzhou is higher than the average value; most of these cities are economically developed and centrally located in a certain region, with good circulation channels for factors and easy spatial association with other cities. The closeness centrality of Anqing, Hefei, Jinhua, and other cities is low. Owing to the limitation of spatial distance and economic development, these cities cannot easily have spatial associations with other cities; therefore, it is necessary to improve their regional synergy.
3. In 2020, the average intermediary centrality of the spatial correlation network of energy-environment efficiency was 5.33, the dominant role of the network center node was strong, and the unbalanced characteristics of the network structure were obvious. Cities such as Shanghai, Nanjing, and Wuxi have a high degree of intermediary centrality. These cities have a strong ability to control the spatial correlation of energy-environment efficiency in other cities, and play obvious “intermediary” and “bridge” roles in the correlation network. The intermediary centrality of Hefei, Anqing, Jinhua, Zhoushan, and other cities is low and the spatial correlation control of other cities is weak.

#### Spatial clustering structure characteristics

**Figure 6:**
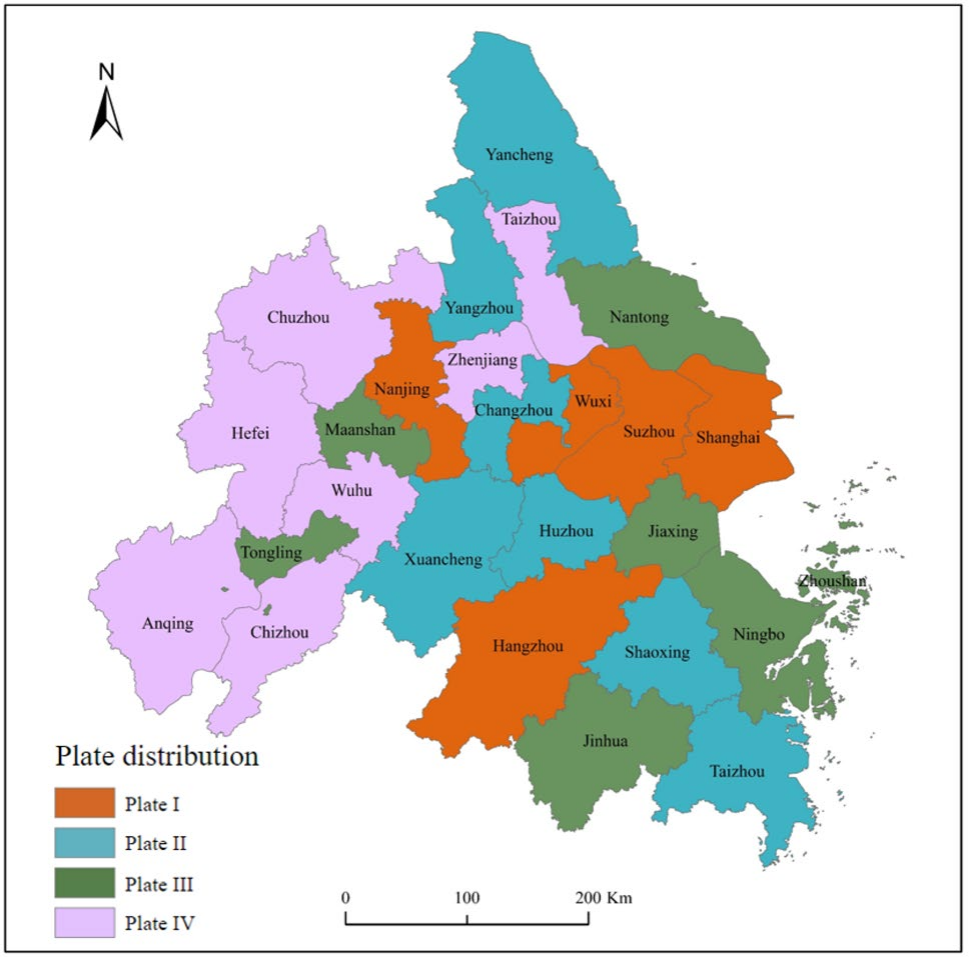
The distribution of cities in the four plates. Authors used Arcgis 10.8 software to draw the map. https://www.arcgis.com/index.html.

With the help of the CONCOR tool in Ucinet 6.0 software, a block model was constructed to analyze the spatial correlation network of energy-environment efficiency in the Yangtze River Delta Urban Agglomeration, which was divided into four plates (Fig. 6), and the description of each plate is shown in Table 6.

**Table 6:** Division of spatial correlation plates of energy-environment efficiency.

Plate	Number of received relationships		Number of sent relationships		Expected internal relationship ratio (%)	Actual internal relationship ratio (%)	Plate role
	Inside	Outside	Inside	Outside			
Plate I	4	70	4	16	16.41	13.19	Net benefit
Plate II	17	22	17	34	48.76	42.72	Two-way spillover
Plate III	13	14	13	42	34.21	16.72	Net overflow
Plate IV	4	14	4	28	12.29	10.67	Agent

In 2020, there were 37 intra-plate relationships in the spatial correlation network of energy-environment efficiency in 2020, accounting for 23.41% of the total number of relationships; 121 extra-plate relationships, accounting for 76.59% of the total number of relationships, indicating the existence of spatial clustering effects and spatial spillover between cities’ energy-environment efficiency. Plate I includes five cities, including Shanghai, Nanjing, and Suzhou, with four intra-plate relationships, 70 incoming and 17 outgoing relationships outside the plate, with a greater proportion of desired than actual internal relationships, and is a “net benefit plate.” Plate II includes seven cities, including Changzhou, Yancheng, and Yangzhou, with 17 intra-plate relationships, 22 incoming and 34 outgoing relationships outside the plate were 22 and the proportion of desired internal relationships is greater than the proportion of actual internal relationships, indicating that the plate has spillover effects both internally and externally, and is a “two-way spillover plate.” Plate III includes seven cities, including Nantong, Jiaxing, and Jinhua, with 13 intra-plate relationships, 14 incoming relationships outside the plate, and 42 outgoing relationships outside the plate. The proportion of desired internal relationships is greater than the proportion of actual internal relationships, which is a “net overflow plate.” Plate IV includes seven cities such as Zhenjiang, Hefei, and Anqing, with 4 relationships within the plate, 14 receiving relationships outside the plate, and 28 outgoing relationships outside the plate. The proportion of expected internal relationships is greater than the proportion of actual internal relationships, indicating that the plate both spills over outwards and receives spillover from other plates and is an "“agent plate".”

**Figure 7:**
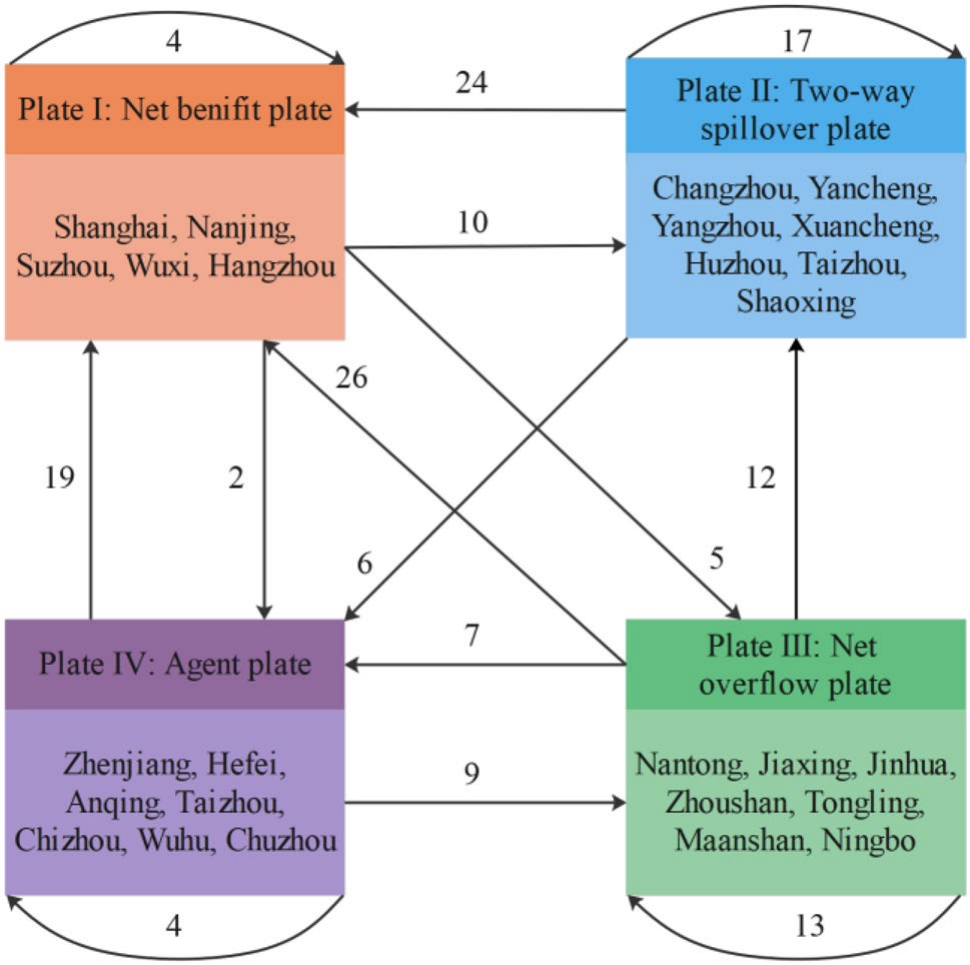
The relationship between the four plates of energy-environment efficiency.

Accordingly, the density matrix of the spatial correlation network of energy-environment efficiency of the Yangtze River Delta Urban Agglomeration was calculated by applying the cohesive subgroup analysis path. The elements of the density matrix that are greater than the network density in 2020 (0.252) are recorded as 1, and the opposite is recorded as 0, to obtain the image matrix (Table 7). Figure 7 depicts the correlation relationship between the plates.

**Table 7:** Density matrix and image matrix of spatial correlation plates of energy-environment efficiency.

Plate	Density matrix				Image matrix			
	Plate I	Plate II	Plate III	Plate IV	Plate I	Plate II	Plate III	Plate IV
Plate I	0.068	0.275	0.129	0.021	0	1	0	0
Plate II	0.462	0.289	0.000	0.257	1	1	0	1
Plate III	0.694	0.352	0.212	0.183	1	1	0	0
Plate IV	0.430	0.000	0.226	0.077	1	0	0	0

Generally, Plate I benefits from the spatial correlation network of energy-environment efficiency by reaping many spillover relationships from other plates because the huge economic size of these cities is highly attractive to low-carbon factors of production such as capital, technology, and talent, which can also lead to polarization between cities. Plate II has spillover effects on both the net benefit plate and the agent plate and accepts the spillover from the net benefit sector; simultaneously, the exchanges within the plate are also more frequent, which fairly regulates the connection between plates, and is a regulator in the network. Plate III has more spillover relationships than receive relationships and is the loser in the network. Plate IV strengthens the connection between the net benefit plate and the net overflow plate through the receiving and spillover relationship and becomes the agent between plates.

### Analysis of driving factors of spatial correlation network

#### QAP correlation analysis

Before the QAP regression analysis, QAP correlation analysis was used to examine the correlation between each explanatory variable and the spatial correlation network matrix of energy-environment efficiency (Table 8).

**Table 8:** Results of QAP correlation analysis and QAP regression analysis.

Variable	QAP correlation analysis		QAP regression analysis	
	Coefficient	*P*-value	Standardized coefficient	*P*-value
*IS*	− 0.358***	0.002	− 0.063*	0.054
*ECS*	0.146*	0.047	0.216	0.223
*ER*	0.319***	0.003	0.357**	0.014
*ED*	− 0.335***	0.002	− 0.751***	0.002
*OU*	− 0.253**	0.042	− 0.085	0.153
*TI*	− 0.282**	0.038	− 0.115**	0.027

Note: *IS*, industrial structure difference matrix; *ECS*, energy consumption structure difference matrix; *ER*, environmental regulation difference matrix; *ED*, economic development difference matrix; *OU*, opening up difference matrix; *TI*, technological innovation difference matrix. *, **, and *** indicate significant at the 10%, 5%, and 1% levels, respectively.

The results of the correlation analysis in Table 8 show that the correlation coefficients of differences in industrial structure, energy consumption structure, environmental regulation, economic development, opening up, and technological innovation with the spatial network structure of energy-environment efficiency are all significantly not equal to zero at the 5% level, indicating that all six factors significantly affect the formation of the spatial correlation network of energy-environment efficiency in the Yangtze River Delta Urban Agglomeration. Among them, the correlation coefficients of the differences in industrial structure, economic development, opening up, and technological innovation are negative, indicating that similar industrial structures and development stages, as well as similar degrees of openness and technology levels, lead to the spatial correlation and spillover of energy-environment efficiency. The correlation coefficient between energy consumption structure differences and environmental regulation differences is positive, indicating that the difference in energy consumption structure and environmental regulation between cities is conducive to the formation of a spatial correlation network of energy-environment efficiency. Additionally, there was some correlation between most of the explanatory variables. Therefore, it was necessary to use QAP regression analysis to reduce the effect of multicollinearity.

#### QAP regression analysis

The results of the QAP regression are shown in Table 8. The overall goodness of fit (R^2^) of the regression equation is 0.583, indicating that the six influencing factors can explain approximately 58.3% of the variation in the spatial correlation network structure of energy-environment efficiency in the Yangtze River Delta Urban Agglomeration. The QAP regression analysis reports unstandardized and standardized regression coefficients. As matrix standardization removes the effect of the magnitude of observations and allows comparison of the degree of influence of different explanatory variables, the standardized regression coefficients are shown directly.

Specifically, the regression coefficient of industrial structure differences is significantly negative at the 10% level, indicating that similar industrial structures between cities have a significant impact on the formation of spatial correlation network of energy-environment efficiency. A similar industrial structure implies a similar stage of development and will to considerably enhance economic and technological links between cities, thus facilitating the formation of correlation network. The difference in the regression coefficient of environmental regulation is significantly positive at the 5% level, indicating that the difference in the strength of government environmental regulation promotes the formation of spatial correlations of energy-environment efficiency. The underlying reason is that cities with relatively strict environmental regulation tend to shift industries to cities with less environmental regulation to some extent, thus promoting the formation of a correlation network of energy-environment efficiency. The regression coefficient of ED is significantly negative at the 1% level, indicating that the smaller the economic development gap between cities, the more conducive it is to the formation of a spatial correlation network of energy-environment efficiency. On the one hand, similar levels of economic development mean that cities are at a similar stage of development and have similar needs for economic growth and environmental improvement, thus strengthening the energy-environment efficiency network; on the other hand, under the action of the market mechanism, production factors are more likely to flow between regions with similar levels of economic development, objectively creating the conditions for the effect of energy-environment efficiency correlation. The regression coefficient of technological innovation differences is significantly negative at the 5% level, indicating that similar technological levels between cities are conducive to the formation of a spatial correlation network of energy-environment efficiency. Similar technological levels are the basis for interregional cooperation and exchange, which facilitates the spillover and absorption of talents and capital, thus stabilizing the network structure. Additionally, the regression coefficients of energy consumption structure differences are positive and those of opening up differences are negative, but neither significant, suggesting that their intercity differences do not significantly affect the formation of energy-environment efficiency networks.

#### Robustness check

This study takes two approaches to carry out robustness tests on the QAP estimates. Firstly, consider selecting several typical years, such as 2010, 2015, and 2020; secondly, change the threshold and build a new distance matrix, such as taking 90% or 110% of the mean as the new threshold. The results of robustness tests are shown in Table 9. The selection of typical years and new distance matrix did not significantly affect the direction and significance of the effects of the explanatory variables, which is generally consistent with the conclusions of the analysis above. This proves the robustness of the QAP regression results.

**Table 9:** Results of robustness tests on the QAP regression.

Variable	Typical year		New threshold	
	2010	2015	90% of the mean	110% of the mean
*IS*	− 0.049*	− 0.055*	− 0.075**	− 0.061*
*ECS*	0.168	0.196	0.243*	0.205
*ER*	0.303***	0.340**	0.408***	0.337**
*ED*	− 0.531**	− 0.669***	− 0.690***	− 0.712***
*OU*	− 0.090	− 0.074	− 0.089	− 0.082
*TI*	− 0.098**	− 0.102**	− 0.128**	− 0.110**

*IS*, industrial structure difference matrix; *ECS*, energy consumption structure difference matrix; *ER*, environmental regulation difference matrix; *ED*, economic development difference matrix; *OU*, opening up difference matrix; *TI*, technological innovation difference matrix. *, **, and *** indicate significant at the 10%, 5%, and 1% levels, respectively.

## Conclusion and discussion

### Conclusions

Based on the scientific measurement of the energy-environment efficiency of 26 prefecture-level cities in the Yangtze River Delta Urban Agglomeration, this study identifies the spatial correlation of energy-environment efficiency among cities through a modified gravity model, uses SNA to reveal the structural characteristics of the spatial correlation network of energy-environment efficiency, and explores the driving factors of the network using the QAP method. The main conclusions of this study are as follows:

1. From 2010 to 2020, the energy-environment efficiency of the Yangtze River Delta Urban Agglomeration generally showed an upward trend, but there were problems of unbalanced and insufficient development. The energy-environment efficiency of most cities has increased significantly, and the gap between cities has narrowed. In terms of spatial distribution, it continues to deepen from the east to the middle and gradually tends to develop in a balanced manner.
2. The spatial correlation of energy-environment efficiency in the Yangtze River Delta Urban Agglomeration shows a more complex network structure. All cities are in the spatial correlation network, the network correlation is increasing, and the network has strong stability. All cities have the potential for external spillover in terms of energy-environment efficiency, with cities such as Shanghai, Nanjing, Suzhou, and Wuxi dominating the network and being the main drivers of energy-environment efficiency; cities such as Anqing, Hefei, and Wuhu are at the periphery of the network.
3. The block model analysis shows that Shanghai and five other cities belong to “net benefit plate” and play a central role in the spatial correlation network of energy-environment efficiency. Seven cities including Changzhou belong to the “two-way spillover plate,” occupying a central position in the network and playing a two-way leading role both inside and outside the plate. Seven cities, including Zhenjiang, are part of the “agent plate” and play a key role in the network, acting as a bridge intermediary. Seven cities, including Nantong, are in the “net overflow plate” and are marginal to the network, playing a “follower” role.
4. Differences in the industrial structure, environmental regulation, economic development, and technological innovation have important implications for the formation of spatial correlation network of energy-environment efficiency. Similar levels of economic development and technology, as well as similar industrial structures, favor the formation of energy-environment efficiency network, while differences in the strength of environmental regulation can facilitate the formation of spatial correlations in energy-environment efficiency.

### Discussions

The findings of the study have important practical implications and policy connotations. First, optimize the spatial correlation network structure of energy-environment efficiency to achieve cross-regional synergy in improving energy-environment efficiency. When developing energy-environment efficiency improvement strategies, the focus should be on not only the size of the attribute data but also the level of the relationship data to gradually form a “quantity-structure” driven cross-regional synergistic improvement mechanism. Second, understand the plate characteristics of the spatial correlation network structure and develop differentiated regional energy-environment efficiency enhancement strategies according to the socioeconomic development characteristics of different plates. For the economically developed “net benefit plate” and “two-way spillover plate”, policy makers should use their technological and management advantages to increase research, development, and use of clean energy; adjust their energy consumption structure; and promote green economic development. For the “net overflow plate” and “agent plate” with a weaker economic base, while maintaining economic growth, strict environmental regulations should be implemented and advanced technology and management tools should be actively introduced to improve energy-environment efficiency. Third, the drivers of spatial correlation network should be comprehensively considered and the two forces of government macro-regulation and micro-market mechanisms should be implemented to promote the spatial linkage of energy-environment efficiency. The government should act to narrow the gap between cities in terms of economic, technological, and industrial development; strengthen the interconnection of energy-environment efficiency between cities; and promote the overall improvement of energy-environment efficiency. Simultaneously, the government should fully utilize market mechanisms such as competition, supply, and demand to strengthen the exchange and cooperation between core and peripheral regions and reduce the spatial unevenness of energy-environment efficiency.

Compared with the existing studies on energy-environment efficiency, this study expands on the perspective and methodology. Practically, this study can provide some useful ideas for solving the problem of energy-environment efficiency: when improving energy-environment efficiency, it is important to consider the impact within and around the region, as well as the network effects across regions. However, there are still some limitations to this study. Referring to the general approach of the existing literature, this study analyses the structure of the spatial correlation network of energy-environment efficiency and its drivers for the most recent year (2020) and draws some conclusions from the study. However, are these structural features and drivers the same across all years? This should be further investigated. Simultaneously, the mechanisms by which each driving factor influences the spatial correlation network need further exploration. In conjunction with future research, this will help in providing comprehensive information and clear directions for achieving synergistic improvements in energy-environment efficiency across regions.

## Data Availability

The datasets used and/or analysed during the current study available from the corresponding author on reasonable request.
